# School Surrounding Region Traffic Commuting Analysis Based on Simulation

**DOI:** 10.3390/ijerph19116566

**Published:** 2022-05-27

**Authors:** Huasheng Liu, Haoran Deng, Yu Li, Yuqi Zhao, Xiaowen Li

**Affiliations:** 1 College of Transportation, Jilin University, Changchun 130022, China; liuhuasheng521@163.com (H.L.); liyu_913@163.com (Y.L.); zhaoyq16@163.com (Y.Z.); lixw1716@mails.jlu.edu.cn (X.L.); 2 College of Engineering, Tibet University, Lhasa 850011, China

**Keywords:** urban traffic, school traffic management, signalized intersection, simulation

## Abstract

Student commuting is an important part of urban travel demand and private car commuting plays an important role in urban traffic, especially in areas near schools. Since parents, especially the parents of elementary and junior high school students, prefer to drive rather than take public transport, there will be a negative effect on traffic management. To address the challenge, a simulation model is established based on schools’ surrounding regions to analyze traffic status. Specifically, the model focuses on urban construction and transportation near the entrance of schools and neighborhoods. In addition, four variable parameters consisting of the directional hourly volume, the parking demand of delivery vehicles, the distance between the school and intersection, and the average parking time for pick-up vehicles are set as influence factors, while traffic efficiency, energy consumption, and pollutant emissions are considered as the evaluation criteria of our model. Extensive simulated experiments show that comparing different scenarios, the traffic state of schools’ surrounding areas can achieve much better performance when the distance between entrances and intersections is 400 m under the 1000 pcu/h condition. This research can provide a scientific basis for school regional traffic management and organization optimization.

## 1. Introduction

With the construction of safe campuses, the government has strengthened the management of vehicle traffic at the entrances and exits of elementary and middle schools. People often utilize relevant models to analyze the relationship between traffic efficiency and multi-parameters. However, because the main traffic flow to schools is usually from the surrounding and marginal areas of the city to the core area of the city [1], commuting to elementary and middle schools has become a difficult issue for urban travel. Studying how school commuting affects traffic and analyzing the main causes of traffic problems can provide a scientific basis for putting forward feasible optimization plans and promoting the construction of safe schools. Hence, precise and effective policies are needed from urban planners to adjust traffic status and maintain the safety of students.

We focus on the effect of school commuting on traffic and expect to find the main influence factors for traffic problems in schools’ surrounding regions. In addition, there are two main challenges that need to be considered when the simulation model is designed. Firstly, there is typically an increased number of vehicles during rush hours. Hence, different from daily public transportation, researchers must propose a policy to allow for more vehicles to pass through at times of increased road use, ensuring smooth highways. Designing an appropriate model to obtain higher traffic efficiency is very challenging. Secondly, driving too slowly often results in more energy consumption, which will lead to increased vehicle exhaust pollutants. Ensuring cars maintain a suitable speed and reducing environmental pollutants is also challenging.

In recent years, with the development of simulation software, VISSIM is one of the most widely used pieces of microscopic traffic software [2], many traffic models have been developed based on VISSIM 7.0 that is developed by PTV company of Germany. Wu et al. [3] established a simulation model of Guangzhou BRT and used the simulation software VISSIM microsimulation platform to evaluate and analyze the impact of Guangzhou BRT on intersections. Chen et al. [4] aimed to effectively model and analyze a highway network based on VISSIM. Xu et al. [5] proposed an optimization scheme and simulated the Baliqiao section at the north latitude of 12th Avenue in Jinan with VISSIM. After the simulation, the optimization evaluation results of the bottleneck section were given. Ali et al. [6] were committed to using Sidra 5 which is developed by ARRB and Transport Research Australia Ltd. intersection software to evaluate and analyze the traffic flow performance of signalized intersections and roundabouts in peak hours in Nicosia City (north of the island). Lownes et al. [7] investigated the impact of driver behavior parameter combination on highway capacity measurement in order to further understand the performance of VISSIM. By studying the results obtained when running the script for the fifth time in COM programming, Istiqomah et al. [8] proposed an effective scheme for an effective method using the interface between PTV VISSIM and external software MATLAB developed by MathWorks, USA. Suthanaya et al. [9] examined an alternative to managing traffic flow using the VISSIM software. In an intersection network without traffic lights, Li et al. [10] solved the two problems of reducing traffic delay and fuel consumption at the same time through a cooperative traffic control algorithm. Cooperative transportation is implemented based on the connection between vehicles and infrastructure (V2I). According to the different attributes of the edge, Zhao et al. [11] determined different edge weights, and then analyzed the statistical characteristics of urban highway traffic networks based on three different network models (a length weighted network model, a traffic capacity weighted network model and a traffic efficiency weighted network model). Wu et al. [12] showed that half of the fuel consumption of the test vehicles was used in the intersection area, while the influence distance and driving time were 28.9% and 68.5%, respectively (nearly 80% was used in the acceleration and idle phases). At the same time, it also showed that the class C traffic condition is the worst way to reduce fuel consumption. After analyzing the above contents, four improvement strategies were put forward. Stepanchuk et al. [13] analyzed the survey of traffic light intersections of the Kyiv Street network. Certain regularities that led to delays and traffic difficulties were revealed in the results obtained. Through the detailed study of rural school transportation in Minas Gerais, Porto et al. [14] proposed a routing method to facilitate rural students to go to school.

In this paper, VISSIM is used to simulate the traffic status of the school’s surrounding region. With changes in directional hourly volume, the parking demand of delivery vehicles, the distance between the school and the intersection, and the average parking time of pick-up vehicles, the influences on traffic operation efficiency, energy, and environmental protection are studied. Through the scene description and numerical analysis, relevant improvement measures are proposed to optimize the traffic operation status of the school area.

## 2. Scene Description and Simulation Modeling

**Figure 1:**
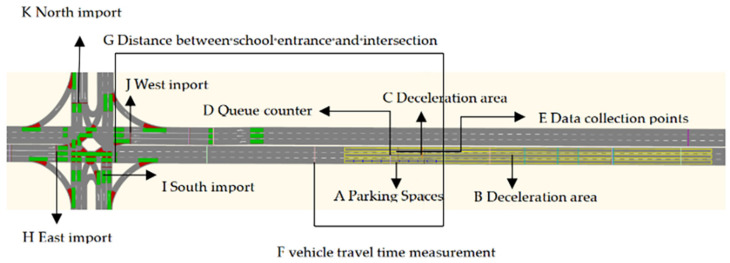
Schematic diagram of simulation scene.

Schools are an important source of generating and attracting transportation, especially during the school commute. Therefore, this paper considers particular school scenarios to build two simulation platforms, and the steps are as follows:

Step 1: Create a network. Urban highways can be divided into four levels: expressway, trunk highway, secondary trunk highway, and branch highway. The forms of urban highway crossing are plane crossing and three-dimensional crossing. Plane crossing includes ring crossing, cross-crossing, T-shaped crossing, dislocation crossing, multi-way crossing, and so on. This paper selects the common trunk highway and trunk highway plane crossing, imports the urban highway base diagram into VISSIM software, and draws the highway network according to it. Each entrance direction is provided with a straight right turn lane, a left turn lane, a straight lane, and a school at the west exit section, as shown in Figure 1.

**Figure 2:**
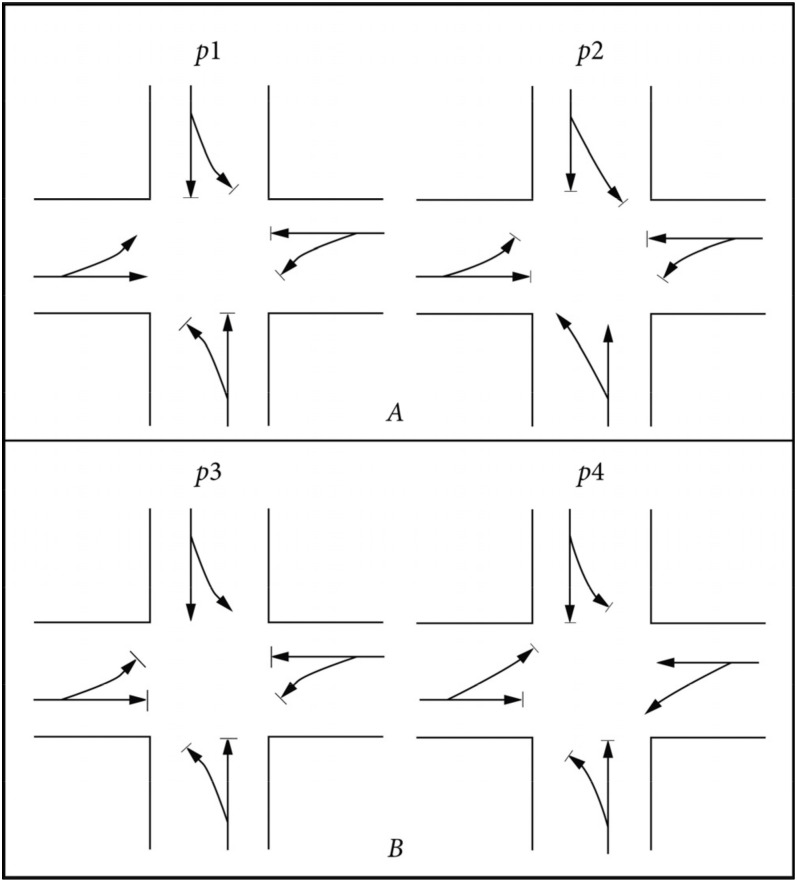
Phase diagram. (**A**) East import and south import phase. (**B**) East import and south import phase.

Step 2: Signal phase setting. The signal phase plan of a typical intersection is determined by the left-turn control, the left-turn phase, and traffic flow [15]. The urban intersection generally adopts two forms of phase, that is, a symmetrical flow release and a separate release at each entrance. In this paper, the separate release at each entrance is adopted, p1 is the east import release phase, p2 is the south import release phase, p3 is the north import release phase, p4 is the west import release phase. As shown in Figure 2.

Step 3: Signal timing. This paper uses the Webster algorithm for signal timing. The timing scheme is based on the change in traffic flow. When the traffic flow is very small, this paper uses the minimum signal cycle.

Minimum Signal Cycle. In this formula, $C_{\min}$ is the minimum intersection duration for a given A-road flow condition minimum signal cycle time; s,j=1,2⋯; $q_{A}\left(j\right)$ is the specific A-way flow rate pcu/h and $q_{BC}\left(j\right)$ is the B-way critical flow rate of the signal to be set under $q_{A}\left(j\right)$ pcu/h [16].

(1) $$C_{\min}=\frac{1.5L+5}{1-\left[q_{A}\left(j\right)/Q_{SA}+q_{BC}\left(j\right)/Q_{SB}\right]}$$

The timing parameters of the Webster Timing Scheme are calculated as follows [17]:

First, the flow ratio sum. In this formula, $Y^{\prime }$ is the individual maximum flow ratio y for all signals comprising the cycle; j is the number of phases in the cycle; $y_{i}$ is the flow ratio of phase j; $q_{b}$ is the design traffic volume pcu/h; and $s_{d}$ is the design saturation flow. When the calculated Y value is greater than 0.9, the inlet channel design or phase design solution must be improved and redesigned [18].

(2) $$Y^{\prime }=\sum_{j=1}^{j}\max\left[y_{i},y_{i}^{\prime },\cdots \right]=\max\left[\left(\frac{q_{d}}{s_{d}}\right),\left(\frac{q_{d}^{\prime }}{s_{d}^{\prime }}\right)\right]$$

Second, the total lost time per cycle. In the equation, $L_{i}$ is the start-up loss time, which should be measured, and is taken when no actual data is available 3 s. $I_{i}$ is the green interval time, i.e., the yellow light time plus the all-red time. $A_{i}$ is the yellow light duration.

(3) $$L=\sum_{i=1}^{n}\left(L_{i}+I_{i}-A_{i}\right)$$

Third, the optimal cycle length. In this formula, L is the total signal loss time; Y is the sum of the flow ratios.

(4) $$C_{0}=\frac{1.5L+5}{L-Y}$$

Fourth, the total effective green light time. In the equation:

(5) $$G_{e}=C_{0}-L$$

Last, the effective green light time for each phase.

(6) $$g_{ej}=G_{e}\frac{\max\left[y_{i},y_{i}^{\prime },\cdots \right]}{Y}$$

Step 4: Priority rule. In this paper, the simulation analysis of motor vehicles is mainly carried out, and the impact of non-motor vehicles and pedestrians at the school surrounding region is not considered. According to the regulations on the implementation of the highway Traffic Safety Law in China, if the turning car meets the straight car, the direct car goes first. If the right-turn car meets the left-turn car, the left-turning car goes first. When traffic overflow occurs, vehicles queue up to enter the exit direction.

**Figure 3:**
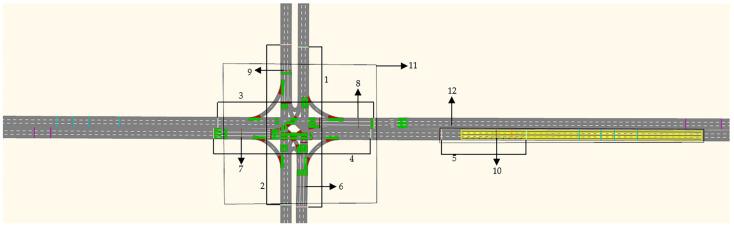
Detector setting.

Step 5: Set detectors. In order to obtain vehicle delay, queue length, vehicle speed, pollutant gas, fuel consumption, and other values, we set up data collection points, vehicle travel time, and node detectors, as shown in Figure 3 and Table 1.

**Figure 4:**
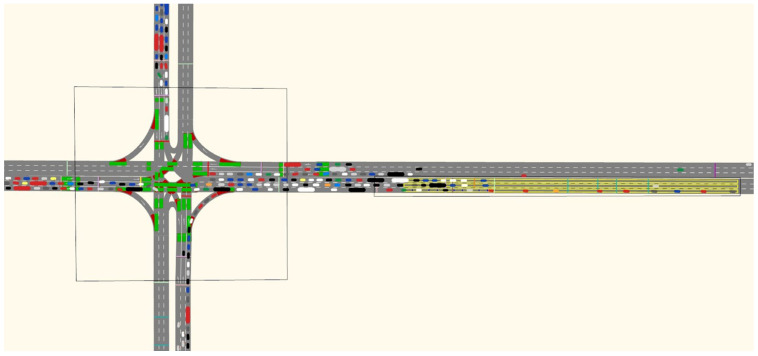
Simulation traffic chart.

Step 6: Simulation configuration. The simulation platform built follows the right-hand driving rules, and the simulation time is 3600 s. Since the simulation preheating takes a certain time, the data between 600 s–3600 s are collected. To ensure that the queue of vehicles did not extend beyond the model, the length of the roadway was set to 1000 m. The green part of the Figure 4 is priority access. Various colors of blotchy are vehicles. The simulation running is shown in Figure 4.

## 3. Simulation Scheme Design and Parameter Setting

### 3.1. Simulation Parameter Setting

The simulation scene of this paper is the bi-directional six-lane intersection of the city, and each entry is set with a left turn lane, a right turn straight lane, and a straight lane. The school entrance is on the west exit, and there are deceleration areas, parking spaces, and stop signs near the school. This paper does not consider the influence of non-motor vehicles and pedestrians on the traffic. Some of the parameter values in the simulation model are taken from the actual survey data of elementary and middle schools, as shown in Table 2. The fixed parameters in the table are unchanged in different simulation scenarios. Variable parameters are used to set different simulation scenarios and are determined according to research needs. The directional hourly volume (DHV) reflects the regional background traffic volume. The parking demand for delivery vehicles (PDD) reflects the size of the school and the arrival rate of transport vehicles during commuting hours. The distance between the school and the intersection reflects the school (DSI) setting scheme in the planning stage. The average parking time of pick-up vehicles (APT) reflects the utilization efficiency of parking spaces.

### 3.2. Analysis Indicators

This paper mainly focuses on the traffic state of the school and intersection areas under the condition of variable parameter changes and analyzes the traffic efficiency, energy, and environment. The indicators are shown in Table 3.

VISSIM is a microsimulation software developed by PTV, Germany, which can generate results to evaluate the service level and operational efficiency of the road network by considering various factors that affect the operation of the road network [19]. The efficiency index represents the smoothness of traffic in different areas (intersections, highway sections in front of schools) within a certain simulation period. This paper analyzes traffic efficiency in five aspects: queue length, vehicle delay, average speed, number of stops, and saturation. This is because the queue length reflects the length of the queued vehicles, the vehicle delay reflects the time lost during the vehicle movement, the average speed reflects the speed of the vehicle movement, the number of stops can characterize the traffic operation status [20], and the saturation reflects the level of congestion on the outlet section. The queue length includes the average queue length and maximum queue length. Vehicle delays include parking delays per vehicle and per vehicle delays. The energy and environmental indicators represent the energy consumption of traffic operation in different regions and the degree of impact on the environment under different variable parameter states during a simulation period. The energy and environment are analyzed in terms of fuel consumption and pollutant gas emissions. Fuel consumption reflects the energy consumption of the vehicle, and pollution gas emissions reflect the environmental impact of the vehicle during operation. In this paper, the 100-km fuel consumption of the vehicle and the vehicle pollutant (CO, NOx, VOC) emissions per unit time are analyzed.

### 3.3. Simulation Scenarios

In this paper, simulation is mainly used for comparative analysis of the following four situations, as shown in Table 4.

In order to clearly describe the influence of DHV, PDD, DSI, and APT on the school’s surrounding region, we set up four different scenarios.

In the first scenario, the impact of DHV on the analysis indexes is studied. The fixed parameters remain unchanged, the change interval of DHV is [100, 2500], the change step is set as 300, and other variable parameters are fixed.

In the second scenario, the impact of PDD on analysis indexes is studied. The fixed parameters remain unchanged, the change interval of PDD is [0, 1000], the change step is set as 100, and other variable parameters are fixed.

In the third scenario, the impact of DSI on the analysis indexes is studied. The fixed parameters remain unchanged, the change interval of DSI is [100, 500], the change step is set as 100, and other variable parameters are fixed.

In the fourth scenario, the impact of APT on analysis indexes is studied. The fixed parameters remain unchanged, the change interval of APT is [10, 60], the step size is set as 10, and other variable parameters are fixed.

## 4. Analysis of Simulation Results

### 4.1. Impact of Directional Hourly Volume on the System

Here, this paper mainly analyzes the results of scenario 1.

#### 4.1.1. Traffic Efficiency

**Figure 5:**
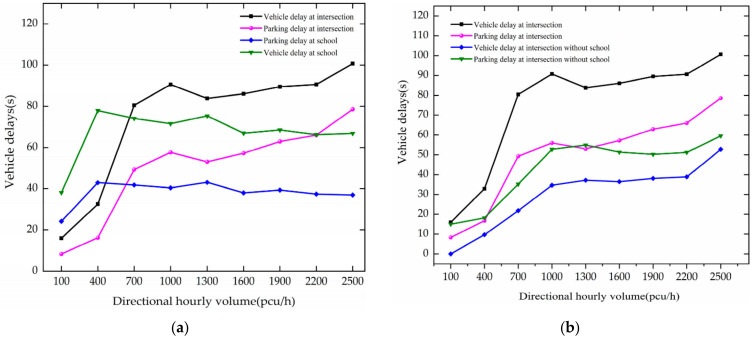
Variation trend of vehicle delay with DHV. (**a**) vehicle delays when there is a school. (**b**) vehicle delays without a school.

Whether to set up a school has a significant difference on the intersection. As shown in Figure 5.

Under the circumstance of there being no school, when DHV is between 100–2500 pcu/h, vehicle delay and stop delay shows a slow increase trend at the intersection. As DHV increases by 300 pcu/h, they increase by 6.6 s and 5.6 s, respectively. After 1000 pcu/h of DHV, they fluctuate around 30.85 s and 53.33 s, respectively.

In the case of a school, when DHV increases at 100–1000 pcu/h, vehicle delay and stop delay increase rapidly at the intersection, and when DHV increases by 300 pcu/h, they increase by 18.69 s and 11.92 s, respectively. After DHV reaches 1000 pcu/h, they fluctuate around 90.13 s and 63.55 s, respectively. In the school’s surrounding region, when DHV increases from 100 to 400 pcu/h, it increases by 39.94 s and 18.78 s, respectively. After 400 pcu/h, both are decreased, which is because parked vehicles choose to pass through the area without parking because parking spaces are occupied.

Through comparative analysis, the school’s surrounding region is more sensitive to the increase in DHV. When there is a school, both increase faster at the intersection. Compared with the fluctuation value, setting up a school will greatly increase the vehicle delay and parking delay at the intersection. The growth in the number of vehicles causes intersections and school zones to quickly reach saturation. The increase in vehicle delays is due to the increased influence of moving vehicles by the preceding vehicles.

**Figure 6:**
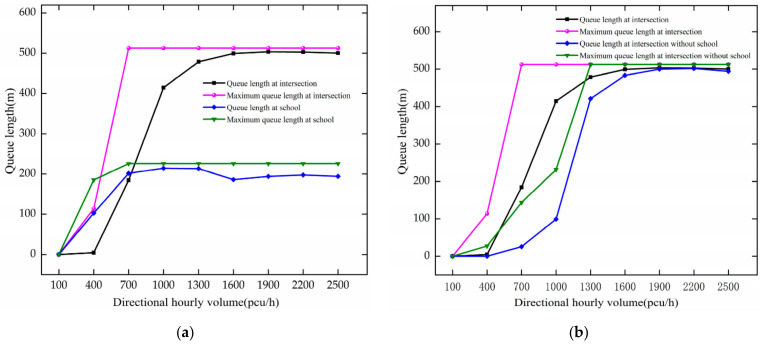
Variation trend of queue length with DHV. (**a**) queue length of the intersection and the school. (**b**) comparison of queue length at the intersection.

The variation trend of queue length with DHV is shown in Figure 6. When there is no school, as DHV increases at 100–1900 pcu/h, the queue length of vehicles increases at the intersection. For every 300 pcu/h increase in DHV, it increases by 71.68 m. After 1300 pcu/h of DHV, it fluctuates around 497.9 m, indicating that the intersection is in a saturation state. The maximum queue length reaches a maximum of 512.40 m at 1300 pcu/h. As the traffic volume increases, the queue length approaches the maximum queue length.

Under the circumstance of a school, as DHV increases at 100–1600 pcu/h, the queue length of vehicles increases rapidly at the intersection. For every 300 pcu/h increase in DHV, it increases by 119.65 m. After DHV reaches 1600 pcu/h, the intersection is saturated, and fluctuates around 501.38 m. The maximum queue length reaches a maximum of 512.40 m at 700 pcu/h. This reaches a maximum faster than when no school is installed, indicating that the installation of a school has a greater impact on the intersection. When DHV increases from 100 to 700 pcu/h in the school surrounding region, they increase by 71.39 m and 75.3 m for every 300 pcu/h increase. After DHV reaches 700 pcu/h, they fluctuate, indicating that the highways near the school are congested.

By comparative analysis, as DHV increases, both queue length and maximum queue length increase. The setting of the school makes them increase more rapidly at the intersection and reach the saturation state faster. The increase in traffic volume increases the queue length at each inlet of the intersection; the school area is affected by the speed limit, etc., which makes the queue length in the school area increase. In the VISSIM software, when the traffic volume increases to the roadway capacity, the number of vehicles does not increase. Therefore, the queues do not get longer above a certain volume of traffic.

**Figure 7:**
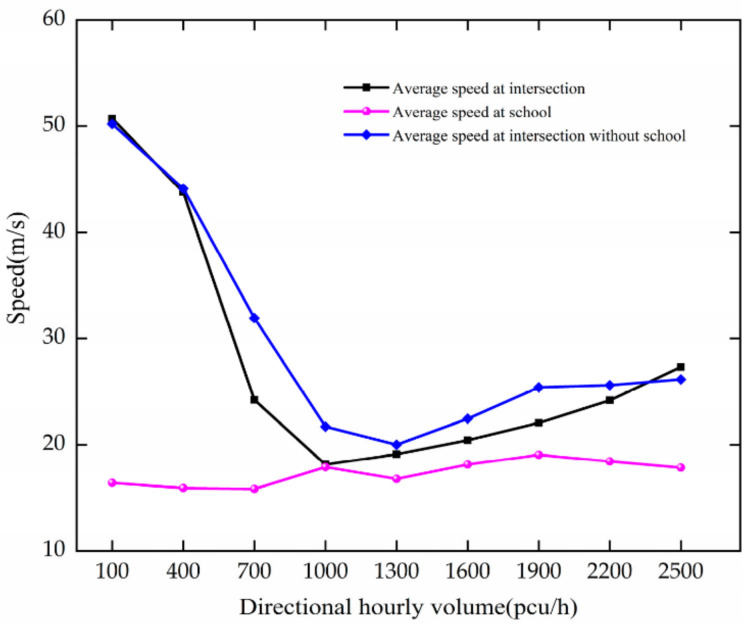
Variation trend of average speed with DHV.

When DHV changes, the variation trend of average speed is as shown in Figure 7. The analysis results are as follows.

Under the circumstance of there being no school, as DHV increases at 100–1300 pcu/h, the average speed of vehicles decreases at the intersection. For every 300 pcu/h increase in DHV, it decreases by 6.02 m/s. After DHV reaches 1300 pcu/h, it fluctuates around 24.91 m/s, indicating that the intersection is in a crowded state.

When there is a school, as DHV increases at 100–1000 pcu/h, the average speed of vehicles at the intersection decreases rapidly. For every 300 pcu/h increase in DHV, it decreases by 10.86 m/s. After DHV reaches 1000 pcu/h, the intersection is in a crowded state, and the average vehicle speed fluctuates around 23.45 m/s. The average speed of vehicles in the school area essentially does not change with an increase in traffic volume, which is due to the installation of a speed reduction zone in front of the school.

By comparative analysis, the increase in DHV reduces the average speed of vehicles at intersections. The average speed decreases faster at intersections with schools. Vehicle speed is affected by the previous vehicle, and vehicles change lanes and other behaviors more frequently at intersections and school areas. Therefore, when traffic flow increases, the average speed of vehicles decreases faster.

**Figure 8:**
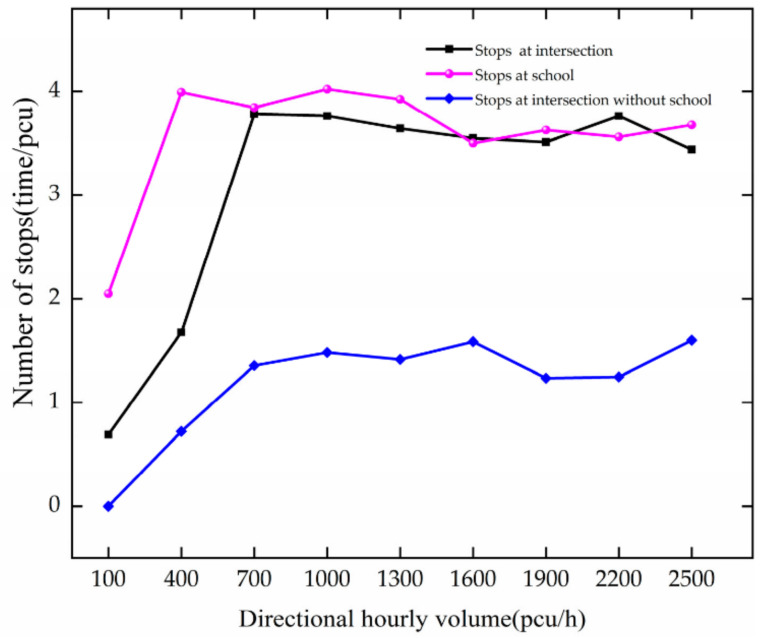
Variation trend of stops with DHV.

The variation trend of stops with traffic volume is shown in Figure 8. When there is no school, as DHV increases from 100 to 1600 pcu/h, the number of stops increases slowly with increasing DHV. The average number of vehicle stops is 1.07.

In the case of a school, as DHV increases at 100–700 pcu/h, the number of stops at the intersection increases rapidly. For every 300 pcu/h increase in DHV, it increases by 1.54 times. The average number of vehicle stops is 2.79. When DHV increases at 100–400 pcu/h, it increases by 1.94 times at the school. After DHV reaches 700 pcu/h, it fluctuates, indicating that the highways near the school are crowded.

The comparative analysis shows that the number of stops at the intersection increases slowly in the absence of schools. When schools are installed, they are more sensitive to changes in DHV and reach congestion more quickly. Schools have a significant impact on the number of stops. The number of vehicles increases, the roads become more congested, and the number of vehicle stops increases with the congestion of the roads.

**Figure 9:**
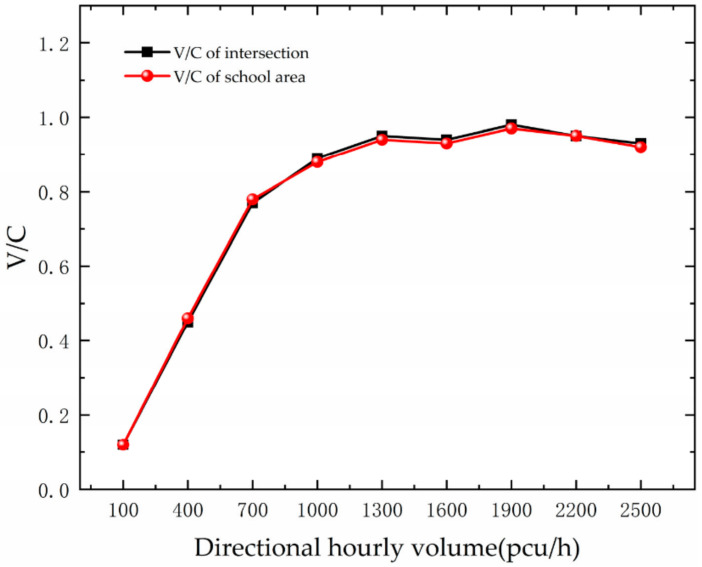
Variation trend of saturation with DHV.

The variation of the intersection and school area saturation with one-way traffic volume is shown in Figure 9. The saturation profiles for the intersection and school areas are essentially the same as the traffic volume increases. At a traffic volume of 1300 pcu/h, congestion is reached, which is also in line with the analysis above.

#### 4.1.2. Energy and Environment

**Figure 10:**
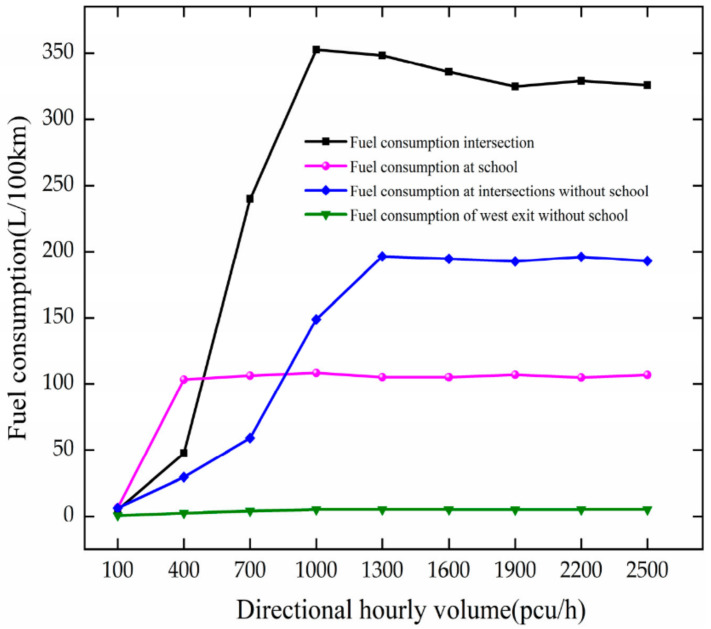
Variation trend of fuel consumption with DHV.

The fuel consumption is analyzed based on Figure 10. The analysis results are as follows.

In the case of no school, as DHV increases at 100–1600 pcu/h, the fuel consumption increases by 57.58 L/100 km for every 300 pcu/h increase. After DHV reaches 1600 pcu/h, it fluctuates around 274.78 L/100 km, indicating that the intersection is saturated.

When there is a school, as DHV increases at 100–1000 pcu/h, the vehicle fuel consumption increases rapidly at the intersection. For every 300 pcu/h increase in DHV, it increases by 115.97 L/100 km. When DHV increases at 100–400 pcu/h, the vehicle fuel consumption increases by 96.96 L/100 km at the school. After 400 pcu/h, it fluctuates near the school, indicating that the intersection is in a crowded state.

Through comparative analysis, the fuel consumption at the intersection and school increase with the growth in DHV. The setting of the school makes the fuel consumption increase more rapidly and reach a state of congestion faster at the intersection. Vehicle delays, queue lengths, number of stops, and increased traffic volume all contribute to increased vehicle fuel consumption.

**Figure 11:**
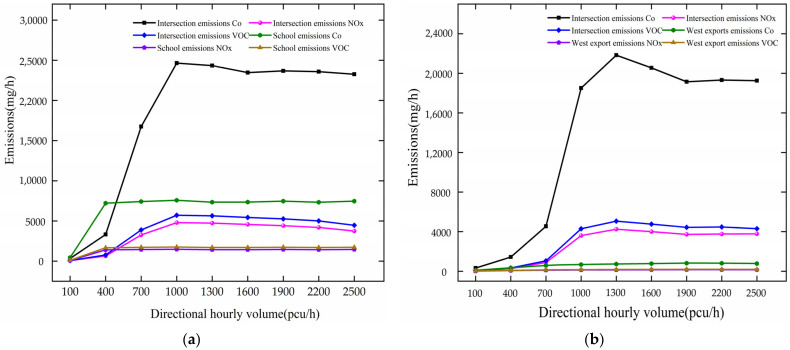
Variation trend of pollutant gas emission with DHV. (**a**) variation trend of pollutants with a school. (**b**) variation trend without a school.

When DHV changes, the variation trend of pollutants is analyzed based on Figure 11. The analysis results are as follows.

Variation trend of pollutant gas emission with DHV is shown in Figure 11. Under the circumstance of there being no school, as DHV increases at 100–1300 pcu/h, the vehicle pollutant gas emission increases at the intersection and west export. For every 300 pcu/h increase, the emissions of CO, NOx, and VOC increase by 5378.72 mg/h, 1046.50 mg/h, and 1246.57 mg/h, respectively, at the intersection; and they are increased by 1246.57 mg/h, 31.08 mg/h, and 37.02 mg/h, respectively, at the west exit section. After DHV reaches 1300 pcu/h, they fluctuate around 19,510.75 mg/h, 3814.44 mg/h, and 4495.82 mg/h, respectively, at the intersection, and they fluctuate around 790.83 mg/h, 153.87 mg/h, and 183.28 mg/h, respectively, at the west exit section.

In the case of a school, as DHV increases at 100–1000 pcu/h, the emissions of CO, NOx, and VOC increase by 8106.52 mg/h, 1577.23 mg/h, and 1878.76 mg/h, respectively, at the intersection. For every 300 pcu/h increase, they increase by 2378.06 mg/h, 462.68 mg/h, and 551.14 mg/h, respectively, at the school. After DHV reaches 1000 pcu/h, they fluctuate around 23,668.07 mg/h, 5160.84 mg/h, and 7392.21 mg/h, respectively, at the intersection, and fluctuate around 7392.21 mg/h, 1438.26 mg/h, and 1713.22 mg/h, respectively, at the school.

Through comparative analysis, the pollutant gas emissions increase at the intersection and school with the growth in DHV, and school pollutant gas emissions are more sensitive to DHV. The school has a great impact on the pollutant gas emission of vehicles at the intersection. Vehicle delays, queue lengths, stopping times, and increased traffic volumes all contribute to the increased vehicle emissions of polluting gases.

### 4.2. The Impact of Parking Demand of Delivery Vehicles on the System

Here, this paper mainly analyzes the results of scenario 2.

#### 4.2.1. Traffic Efficiency

**Figure 12:**
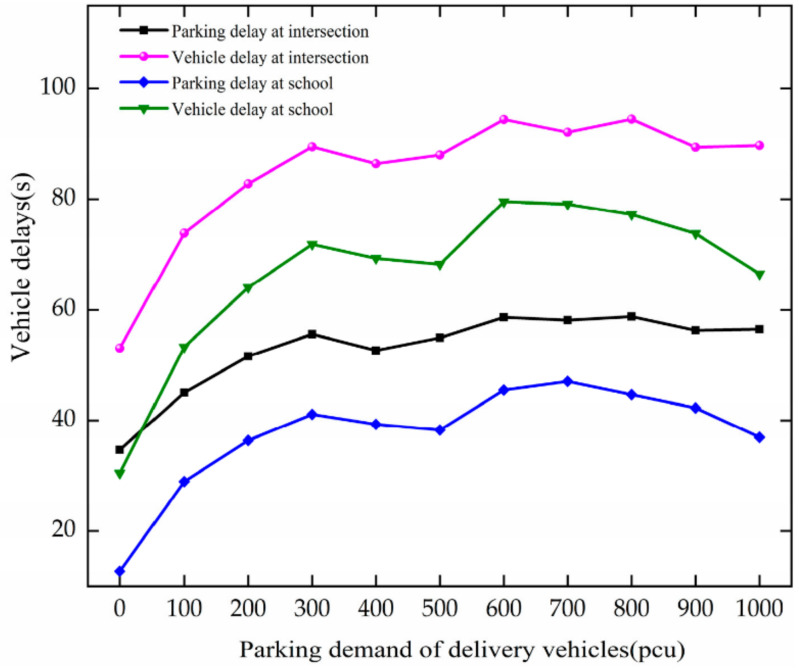
Variation trend of vehicle delay with PDD.

When PDD changes, the trend of vehicle delay is as shown in Figure 12. The analysis results are as follows.

Vehicle delay and parking delay increase with the growth in PDD at the intersection. When PDD increases from 0 to 300 pcu, the speed increases. For every 100 pcu increase, they increase by 6.97 s and 12.12 s, respectively.

The vehicle delay and parking delay increase with the increase in PDD at the school. As PDD increases from 0 to 300 pcu, the speed increases. For every 100 pcu increase, they increase by 9.46 s and 13.79 s, respectively.

Through comparative analysis, with the growth in PDD, vehicle delay and parking delay increase at the intersection and the school, and they increase greatly at the intersection. After the stopping vehicles enter the school area section, most of them need to change lanes and then stop, resulting in increased vehicle delays in the school area section.

**Figure 13:**
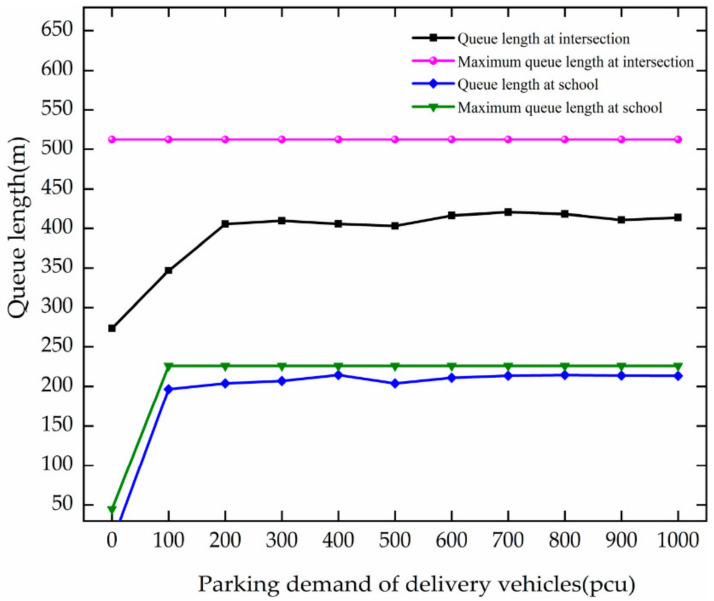
Variation trend of queue length with PDD.

The trend of queue length with the change in PDD is analyzed based on Figure 13. The analysis results are as follows.

In the case of PDD changes from 0 to 200 pcu, the queue length of vehicles increases rapidly at the intersection. For every 100 pcu growth, it increases by 65.87 m. After the PDD reaches 200 pcu, it fluctuates around 412.06 m. The maximum queue length is basically unchanged because it has been reached at the traffic volume of 1000 pcu/h.

When the PDD increases from 0 to 100 pcu, the queue length and the maximum queue length increase by 193.21 m and 181.21 m, respectively, in the school’s surrounding region. After the PDD reaches 100 pcu, the queue length and the maximum queue length fluctuate around 210.59 m and 225.90 m, respectively.

By comparative analysis, when the PDD varies from 0 to 200 pcu, the queue length of vehicles increases with the PDD at the intersection; when the PDD varies from 0 to 100 pcu, the queue length and maximum queue length of vehicles increase with the PDD at the school area. The increased demand for vehicle stops and frequent vehicle lane changes lead to increased queue lengths. After the number of parked vehicles is 100, the parking spaces are occupied, vehicles cannot be parked, and queue length fluctuates and changes.

**Figure 14:**
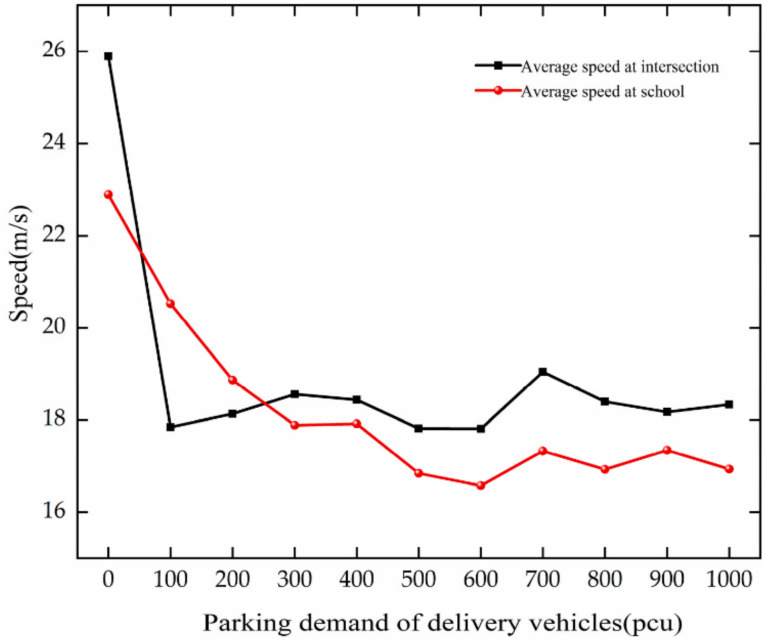
Variation trend of average speed with PDD.

As PDD changes, the trend of average speed is analyzed based on Figure 14. The analysis results are as follows.

The average speed of vehicles decreases with the growth in PDD at the intersection. When PDD increases from 0 to 100 pcu, the speed decreases faster. It decreases by 8.05 m/s.

The average speed at the school decreases with the growth in PDD. When PDD increases from 0 to 300 pcu, the speed decreases faster. For every 100 pcu increase, it decreases by 2.01 m/s.

Through comparative analysis, with the growth in PDD, the average speed of vehicles decreases at the intersection and the school. The reduction is large at the intersection. Vehicle speed is mainly affected by the driving status of the previous vehicle, and the increase in the number of stops leads to frequent vehicle changes, which makes the average speed of the vehicle further decrease.

**Figure 15:**
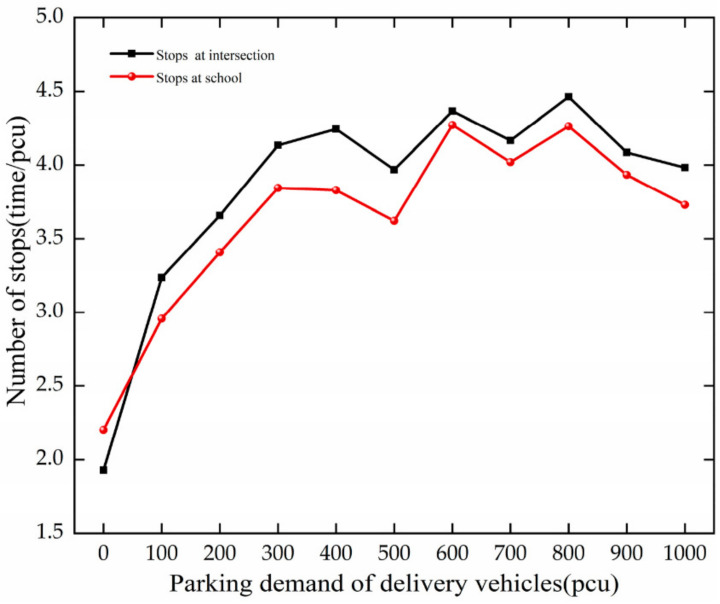
Variation trend of stops with PDD.

The trend of stops is analyzed based on Figure 15. The analysis results are as follows.

The stops increase with the growth in PDD at the intersection and the school. When PDD increases from 0 to 300 pcu, there is an increasing speed of stops. For every 100 pcu increase, they increase by 0.73 and 0.55, respectively, at the intersection and the school.

Through comparative analysis, with the increase in PDD, the stops of vehicles increase at the intersection and the school. The growth of vehicle stops at the intersection is larger than at the school. The increase in the number of parking lots has led to increased congestion and parking at the school.

**Figure 16:**
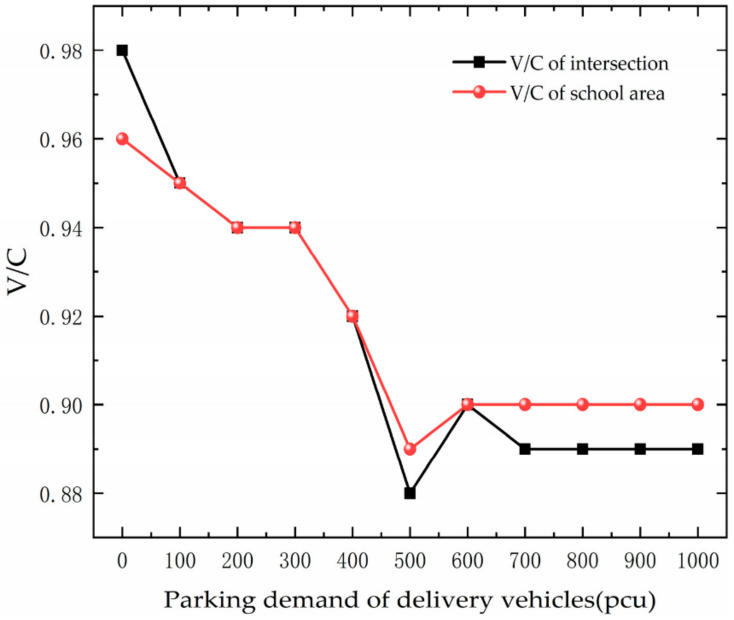
Variation trend of Saturation with PDD.

The trend of saturation is analyzed based on Figure 16. As the PDD changes, the saturation of the intersection and school area first decreases, and then the area remains unchanged. This is because as the PDD increases, it reduces the number of vehicles that can access the roadway. The limited number of parking spaces leads to the fact that vehicles have a demand for parking but are unable to stop, so the saturation of the road section no longer changes.

#### 4.2.2. Energy and Environment

**Figure 17:**
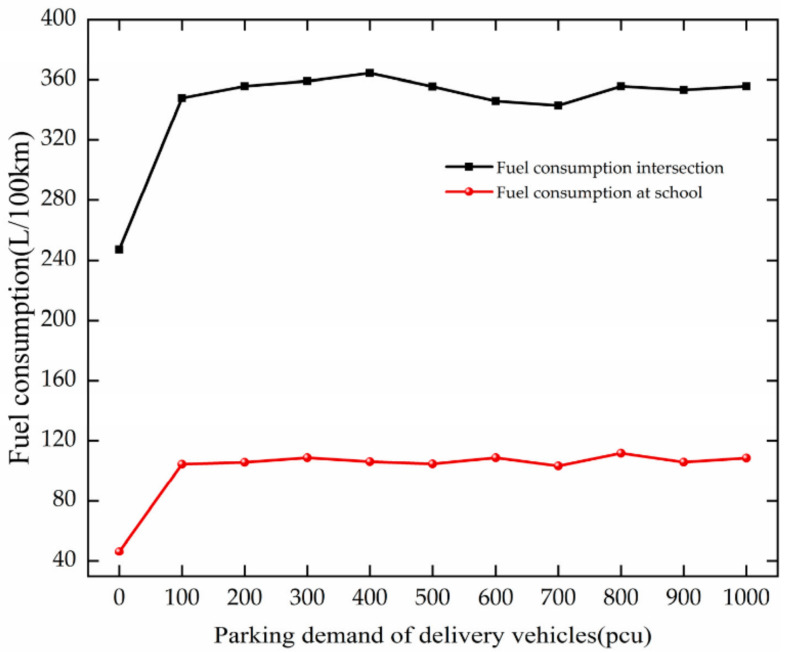
Variation trend of fuel consumption with PDD.

The trend of fuel consumption is analyzed based on Figure 17. The fuel consumption of vehicles increases with the increase in PDD at 0–100 pcu at the intersection and the school. They increase by 100.54 L/100 km and 58.13 L/100 km, respectively, at the intersection and school. After PDD reaches 100 pcu, they fluctuate around 398.72 L/100 km and 120.53 L/100 km, respectively, at the intersection and the school.

**Figure 18:**
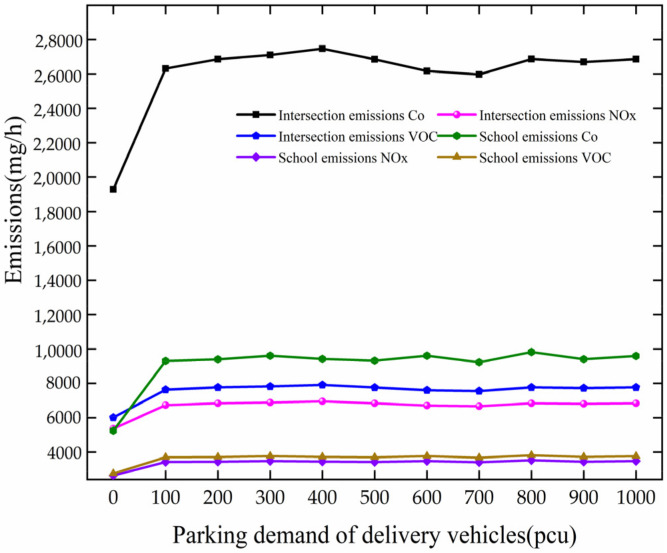
Variation trend of pollutant gas emission with PDD.

Analysis of the pollutant gas emission is based on Figure 18. When PDD increases from 0 to 100 pcu at the intersection, the growth rate of pollutant gas emissions is increased. The emissions of CO, NOx, and VOC increase by 7027.54 mg/h, 1367.30 mg/h, and 1628.70 mg/h, respectively, at the intersection. After PDD reaches 100 pcu, the emission of pollutant gases fluctuates.

As PDD changes from 0 to 100 pcu at the school, the speed increases more slowly. They increase by 4063.05 mg/h, 790.52 mg/h, and 941.65 mg/h, respectively, at the school. After PDD reaches 100 pcu, the emission of pollutant gases fluctuates.

Through comparative analysis, as PDD increases from 0 to 100 pcu, the emission of pollutant gases increases rapidly at the intersection and the school. After PDD reaches 100 pcu, they fluctuate around 27,856.34 mg/h, 5419.82 mg/h, 6455.98 mg/h, 8425.19 mg/h, 1639.24 mg/h, and 1952.62 mg/h, respectively, at the intersection and the school.

### 4.3. The Impact of Distance between School and Intersection on the System

Here, we mainly analyze the results of scenario 3.

#### 4.3.1. Traffic Efficiency

**Figure 19:**
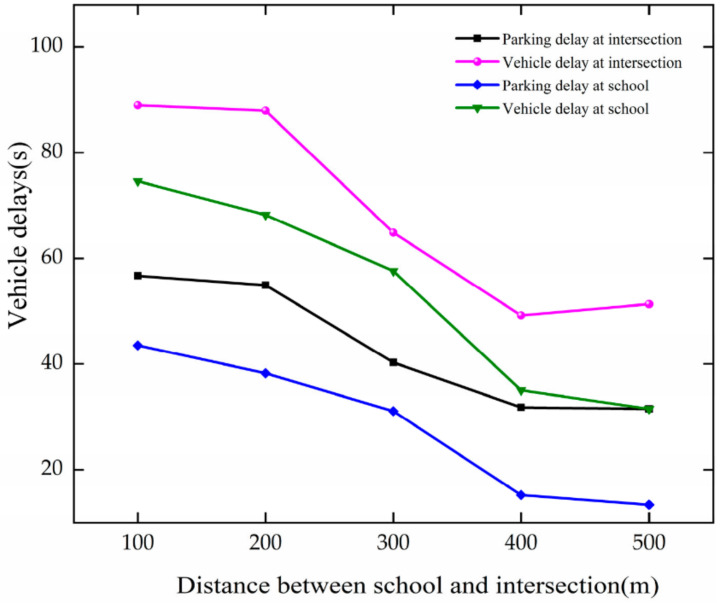
Variation trend of vehicle delay with DSI.

As DSI changes, the trend of vehicle delay is analyzed based on Figure 19. Vehicle delay and parking delay decrease with the growth in DSI at the intersection and the school. When DSI increases between 100–400 m, they decrease faster at the intersection. For every 100 m growth in DSI, they decrease by 9.31 s, 13.26 s, 9.43 s, and 13.20 s, respectively, at the intersection and school. The results show that vehicle delay and parking delay decrease with the increase in DSI at the intersection and the school. After the DSI reaches 400 m, they fluctuate around 31.65 s, 50.27 s, 14.29 s, and 33.28 s, respectively, at the intersection and school. The increased distance between the school and the intersection results in less interaction between the school area and the intersection, and thus less vehicle delay.

**Figure 20:**
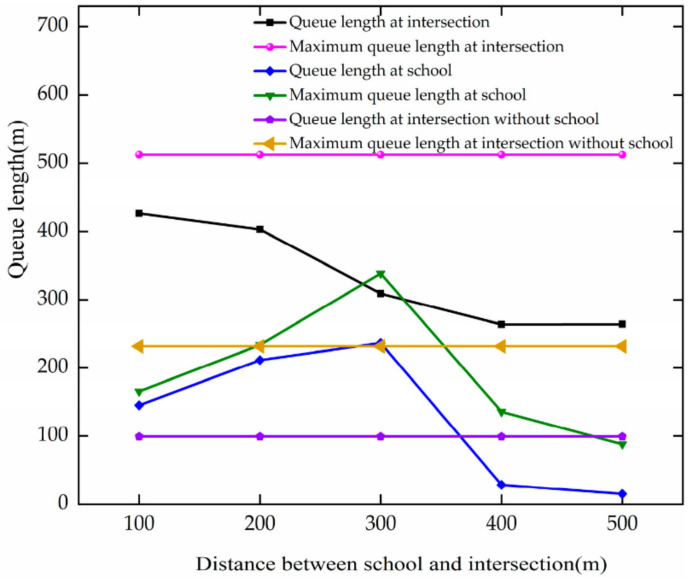
Variation trend of queue length with DSI.

The trend of queue length is analyzed based on Figure 20. The analysis results are as follows.

The maximum queue length of vehicles at the intersection does not change much when the DSI changes, since the maximum queue length has already been reached at the intersection at a traffic flow of 1000 pcu/h. When the DSI increases at 100–400 m, the queue length decreases by 163.13 m. For every 100 m increase, there is a 54.38 m reduction.

The vehicle queue length and the maximum queue length at the school increase with the change in DSI from 100 to 200 m. They increase by 45.74 m and 86.45 m, respectively, at the school for each 100 m increase. In the case of the DSI change from 200 m to 400 m, the vehicle queue length and maximum queue length decrease at the school. They decrease by 207.94 m and 202.51 m, respectively, at the school.

The comparative analysis shows that the queue length and maximum queue length decrease as the DSI of the intersection increases, but increase as the DSI increases from 100 to 300 m at the school. When the DSI increases to 400 m, the queue length and maximum queue length up to both the school area and intersection are smaller. As the DSI increases, the impact of the school zone on the intersection decreases, and the intersection vehicle queue length decreases. The school area queue length increases and then decreases because the traffic characteristics of the school area show up as the DSI increases. When the DSI increases to 400 m, the vehicle speed in the school area increases, and the capacity increases, making the vehicle queue length decrease.

**Figure 21:**
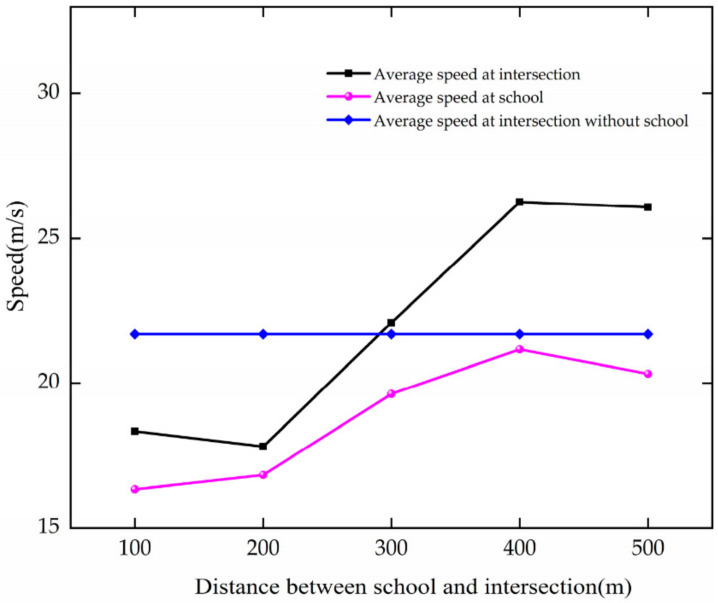
Variation trend of average speed with DSI.

The analysis of the average speed is based on Figure 21, and the results are as follows.

In the case of DSI increases from 100 to 400 m, the average speed of vehicles increases at the intersection and school. For every 100 m increase, they increase by 2.64 m/s and 1.61 m/s, respectively.

The results show that when DSI increases from 100 to 400 m, the average speed of vehicles increases at the intersection and the school. With the increase in DSI, the impact of the school on the intersection can be reduced. When the DSI increases to 400 m, the average speed of vehicles in the school area and at the intersection reaches a better state.

**Figure 22:**
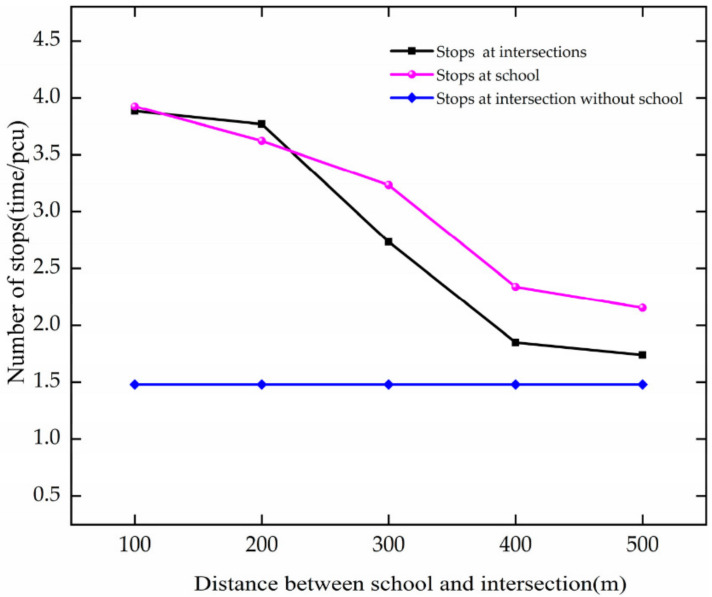
Variation trend of stops with DSI.

The trend of stops is analyzed based on Figure 22. The analysis results are as follows:

In the case of DSI increases from 100 to 400 m, the number of stops decreases at the intersection and school. For every 100 m growth, they decrease by 0.68 and 0.53, respectively.

Through comparative analysis, when DSI increases from 100 to 400 m, the number of stops decreases at the intersection and the school. With the growth in DSI, the impact of the school on the intersection is reduced. As the DSI increases, the average speed of the vehicle increases, indicating that the vehicle is moving more smoothly, so the number of stops decreases.

**Figure 23:**
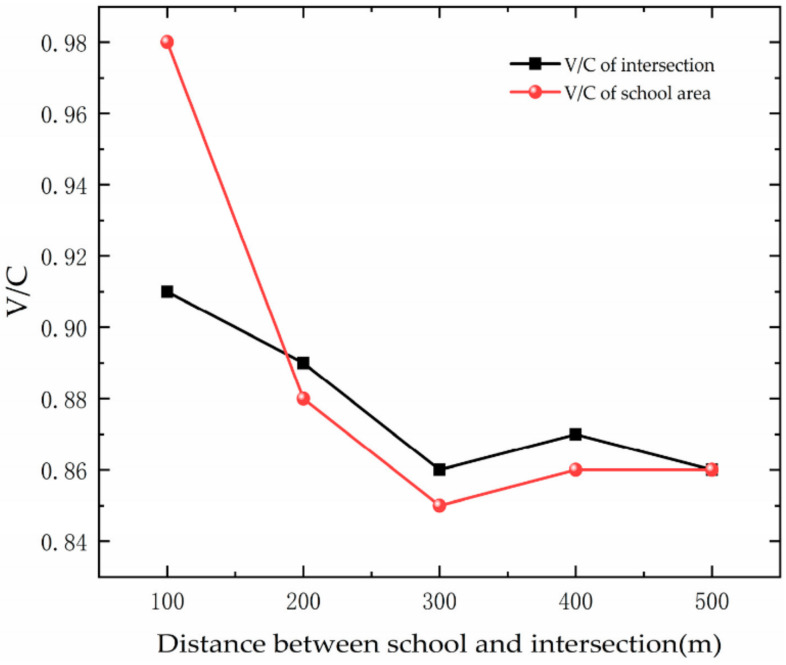
Variation trend of Saturation with DSI.

Figure 23 shows the trend of saturation with DSI. As the DSI increases, the saturation of the road decreases. This is because as the DSI changes, the capacity is affected by several factors and changes non-linearly, while the saturation of intersections and school sections decreases with a certain amount of traffic.

#### 4.3.2. Energy and Environment

**Figure 24:**
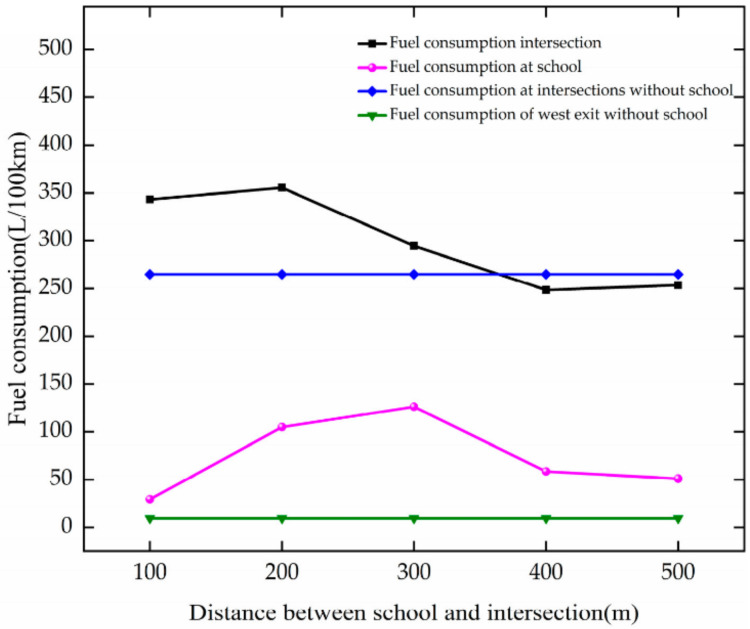
Variation trend of fuel consumption with DSI.

As DSI increases, the trend of fuel consumption is analyzed based on Figure 24. The analysis results are as follows.

When DSI increases from 100 to 400 m, the fuel consumption of vehicles decreases at the intersection. For every 100 m growth in DSI, it decreases by 23.73 L/100 km.

In the case of DSI increases from 100 to 300 m, it increases at the school. For every 100 m growth of DSI, it increases by 48.43 L/100 km. With the increase in DSI from 300 to 400 m, it reduces by 67.69 l/100 km.

The results show that fuel consumption decreases with the growth in DSI at the intersection. When DSI increases to 400 m, the impact of setting up a school on the intersection is reduced and the fuel consumption of vehicles in the school area reaches a better state.

**Figure 25:**
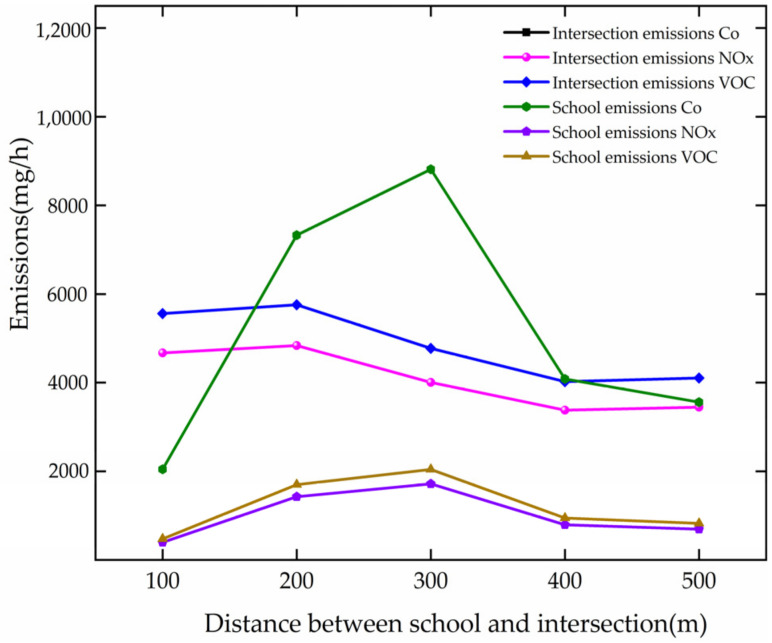
Variation trend of pollutant gas emission with DSI.

The analysis of the pollutant gas emission is based on Figure 25, and the results are as follows.

The pollutant gas emissions decrease with the growth in DSI at the intersection. For every 100 m increase, the emissions of CO, NOx, and VOC are reduced by 2211.30 mg/h, 430.24 mg/h, and 512.49 mg/h, respectively, at the intersection.

When the DSI increases from 100 to 300 m, they increase at the school. For every 100 m growth of DSI, they increase by 3385.00 mg/h, 658.60 mg/h, and 784.51 mg/h, respectively, at the school. When the DSI increases from 300 to 400 m, they decrease by 4731.74 mg/h, 920.62 mg/h, and 1096.63 mg/h, respectively, at the school.

The comparative analysis shows that the pollutant gas emissions at the intersection decrease when the DSI increases from 100 to 400 m. When the DSI changes to 400, they reach a better state of emissions at schools and intersections.

### 4.4. Impact of Average Parking Time for Pick-Up Vehicles on the System

Here, we mainly analyze the results of scenario 4.

#### 4.4.1. Traffic Efficiency

**Figure 26:**
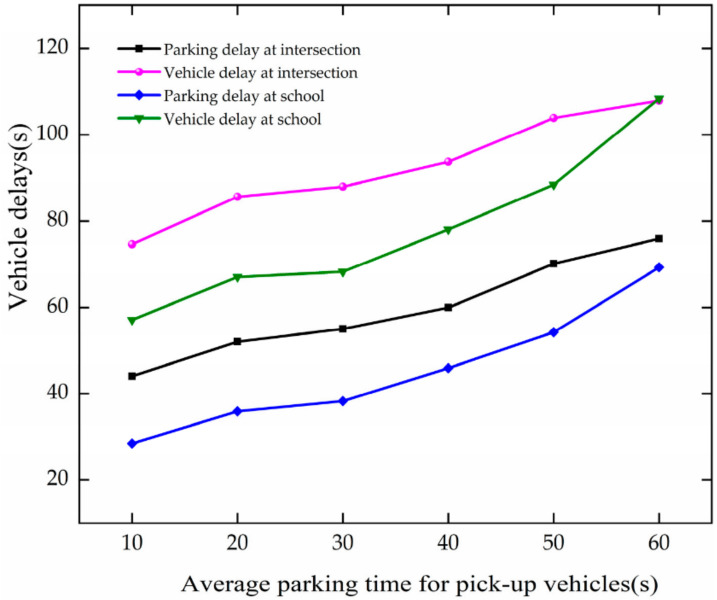
Variation trend of vehicle delay with APT.

In the case of APT changing, the trend of vehicle delay is analyzed based on Figure 26. The analysis results are as follows.

The vehicle delay increases with the growth of APT at the intersection and school. When APT increases from 10 to 60 s, the vehicle delay and parking delay increase by 6.38 s, 6.66 s, 8.16 s and 10.27 s, respectively, at the intersection and school. The results show that vehicle delay and parking delay increase with the growth in APT at the intersection and the school. They increase more rapidly at the school. The increase in stopping time leads to more congestion on the roadway, causing increased vehicle delays.

**Figure 27:**
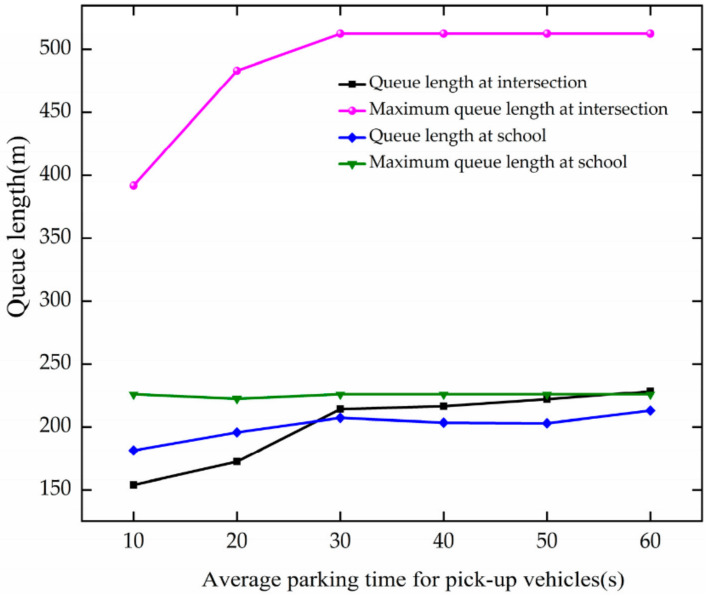
Variation trend of queue length with APT.

The trend of queue length is analyzed based on Figure 27. When APT increases at 10–30 s, the queue length, and maximum queue length increase at the intersection. For every 10 s growth of APT, they increase by 30.05 m and 60.27 m, respectively. The maximum queue length does not change with the increase in APT at the school. As APT increases at 10–30 s, the queue length increases at the school. For every 10 s increase in APT, it increases by 12.91 m.

Through comparative analysis, the queue length and maximum queue length increase with the growth of APT at the intersection, and the increased speed is faster than the queue length of the school. When APT reaches 30 s, they fluctuate around 220.06 m, 512.40 m, 206.54 m, and 225.79 m, respectively, at the intersection and school.

**Figure 28:**
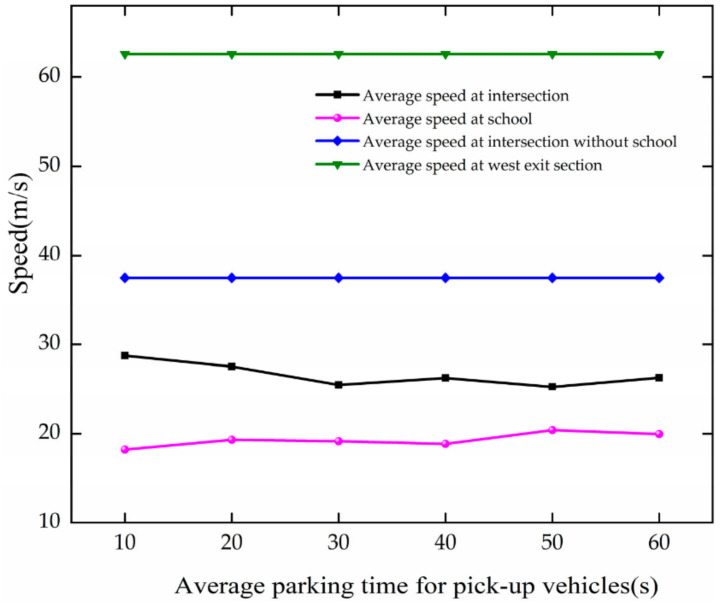
Variation trend of average speed with APT.

The analysis of average speed is analyzed based on Figure 28, and the results are as follows.

The average speed is basically unchanged with the growth of APT at the intersection and the school. They fluctuate around 26.59 m/s and 19.32 m/s, respectively, at the intersection and school. Since the road is more congested and the average vehicle speed is lower at a one-way traffic volume of 1000 pcu/h, the increase in vehicle stopping time has less effect on the average speed.

**Figure 29:**
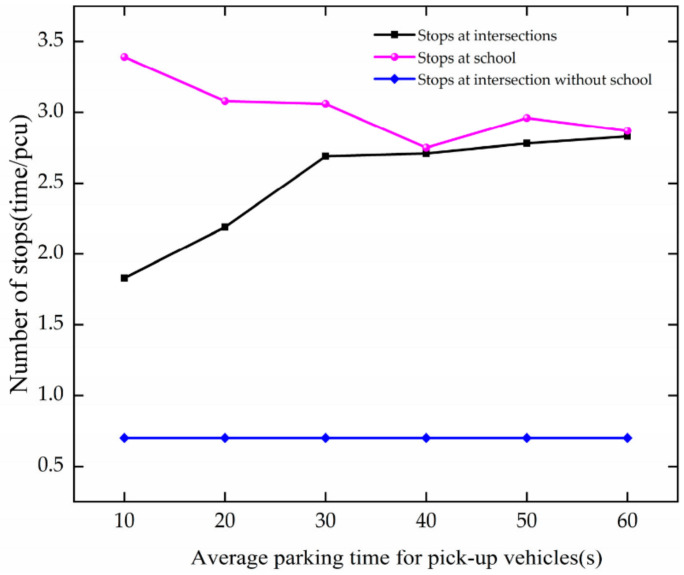
Variation trend of stops with APT.

The trend of stops is analyzed based on Figure 29. The analysis results are as follows.

In the case of APT increases at 10–30 s, the number of stops increases at the intersection. For every 10 s growth of APT, it increases by 0.43. As APT increases at 10–40 s, it decreases at the school. For every 10 s increase in APT, it decreases by 0.21. This is because parking time increases, parking spaces are occupied, and vehicles pass through directly without stopping.

The results show that when APT is increased within 10–40 s, the number of stops at the intersection increases due to vehicle decisions that require stopping in the school zone, and the number of stops at the school decreases due to occupied parking spaces in the school zone that prevent vehicles from stopping, allowing them to pass through directly.

**Figure 30:**
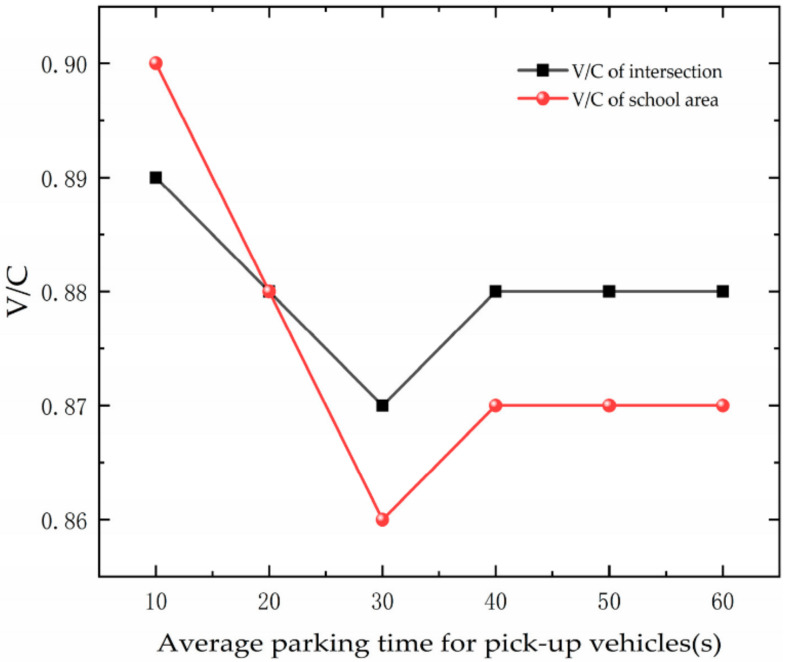
Variation trend of saturation with APT.

Figure 30 shows the variation trend of saturation with APT. As the APT increases, the saturation of the road first decreases and then remains essentially the same. The increase in vehicle stopping time decreases the roadway capacity while causing an increase in parking space occupancy time. This results in vehicles with a need to stop being unable to stop. School area road capacity is affected by several factors, including vehicle stopping times, and shows non-linear variations, while traffic volumes remain largely constant.

#### 4.4.2. Energy and Environment

**Figure 31:**
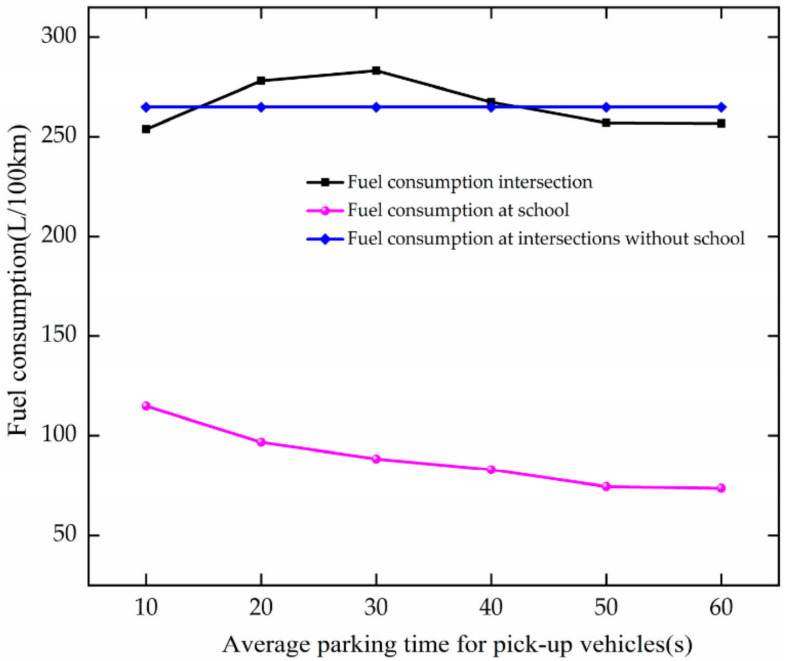
Variation trend of fuel consumption with APT.

As APT increases, the trend of fuel consumption is analyzed based on Figure 31. When APT increases at 10–20 s, the fuel consumption increases at the intersection. It increases by 24.35 m. This is due to the increase in vehicle stopping time, which makes the school entrance set-up have an increased impact on the intersection. When APT increases at 30–40 s, it decreases. The fuel consumption of vehicles in school zones decreases with increased APT. This is because the parking time of vehicles increases, and the occupying time of parking spaces increases. Many vehicles cannot stop and pass through directly.

**Figure 32:**
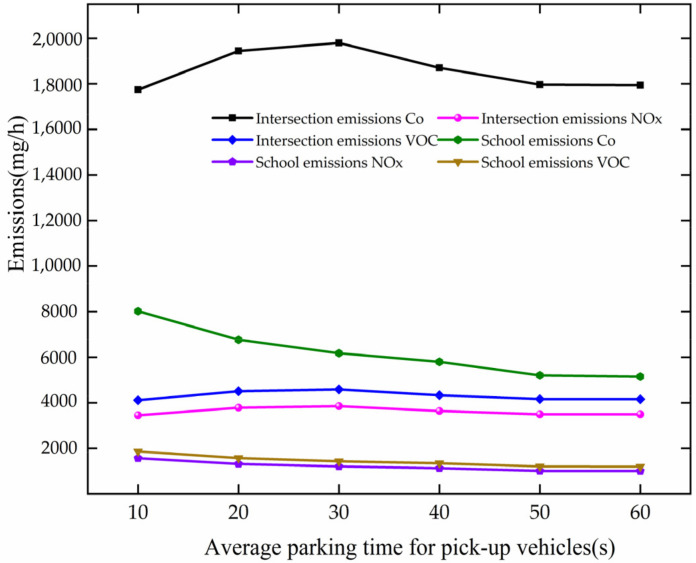
Variation trend of pollutant gas emission with APT.

The trend of pollutant gas emission is analyzed based on Figure 32. The trend in emissions of polluting gases is approximately the same as the trend in fuel consumption, which is also due to many vehicles which cannot stop and pass through directly.

## 5. Conclusions

VISSIM is used to simulate the adjacent signal-controlled intersection upriver of the school. The school is set at the west exit of the urban trunk highway intersection as an example; it is mainly evaluated from the perspective of highway traffic efficiency, energy, and environment. The research shows that when DHV increases, vehicle queue lengths, vehicle delays, stopping times, fuel consumption, and pollutant gas emissions increase in the school and intersection areas, which is roughly the same trend as with roadway saturation. This is due to the growth in the number of vehicles, which causes the road to reach congestion. When the PDD increases, the analysis indicator will rise and then remain unchanged except for the average speed. This is because vehicle stopping time increases, resulting in a decrease in roadway capacity and a rise in congestion. However, when the number of stopped vehicles increases to a certain number, the number of parking spaces cannot meet the demand and no longer has an impact. When the DSI increases to 400 m, the indicators in the school and intersection area reach a better state. When the APT changes, most of the indicators first show an upward trend then the area stabilizes; this is because when the APT increases to a certain value, the parking space is occupied and vehicles with parking needs cannot park.

## Figures and Tables

**Table 1:** Detector classification.

Serial Number	Detector Type	Evaluation Index
1, 2, 3, 4, 5	vehicle travel time	vehicle delays
6, 7, 8, 9, 10	data collection points	average speed
11, 12	node	fuel consumption and pollutant gas

**Table 2:** Parameter setting of simulation platform.

Parameters Class	Name of Parameter	Units	Value/Range/Description
highways and intersection	number of one-way lanes	lane	3
	lane width	m	3.5
	number of parking space	pax	10
	length of parking space	m	6
proportion of traffic volume of each lane and each flow direction entering the school section	east import	-	0.8
	west import	-	0.3
	south import	-	0.45
	north import	-	0.41
traffic flow	the proportion of cars	%	90
	the proportion of buses	%	10
	acceleration and deceleration	$m/s^{2}$	−4
signal timing dial	east import	s	57
	west import	s	50
	south import	s	42
	north import	s	47
	yellow light time	s	3
control parameters	school zone speed limit	km/h	30
	highway speed limit	km/h	60
variable parameter	directional hourly volume	pcu/h	100–2500
	parking demand of transport vehicles	pcu	0–600
	distance between school and intersection	m	50–500
	average parking time for pick-up vehicles	s	10–60

**Table 3:** Analysis indicators setting.

Indicator Categories	Name of Index	Units
traffic efficiency	queue length	m
	vehicle delays	s
	average speed	m/s
	number of parking	time/car
	road section saturation	-
energy and environment	fuel consumption	L/100 km
	emission of air pollutants	mg/h

**Table 4:** Parameter setting of simulation scenario.

Variable Parameter	Units	Scenario 1	Scenario 2	Scenario 3	Scenario 4
Directional hourly volume (DHV)	pcu/h	[100, 2500]	1000	1000	1000
Parking demand of delivery vehicles (PDD)	pcu	500	[0, 1000]	500	500
Distance between school and intersection (DSI)	m	200	200	[50, 500]	200
Average parking time for pick-up vehicles (APT)	s	30	30	30	[10, 60]

## Data Availability

Not applicable.
